# Prevalence of voluntary dehydration according to urine osmolarity in elementary school students in the metropolitan region of São Paulo, Brazil

**DOI:** 10.6061/clinics/2019/e903

**Published:** 2019-09-19

**Authors:** Francine C Dias, Sabine N Boilesen, Soraia Tahan, Lígia CFL Melli, Mauro B Morais

**Affiliations:** Divisao de Gastroenterologia Pediatrica, Escola Paulista de Medicina, Universidade Federal de Sao Paulo, Sao Paulo, SP, BR

**Keywords:** Prevalence, Voluntary Dehydration, Children, Urine Osmolarity, Nutritional Status

## Abstract

**OBJECTIVES:**

To evaluate the prevalence of voluntary dehydration based on urine osmolarity in elementary school students from two public educational institutions in the metropolitan region of São Paulo and evaluate whether there is a relationship between voluntary dehydration and nutritional status or socioeconomic status.

**METHODS:**

Analytical cross-sectional study with students from two public schools in the city of Osasco. The determination of urine osmolarity was performed using the freezing method of the Advanced® Osmometer Model 3W2. Urine osmolarity greater than 800 mOsm/kg H_2_O was considered voluntary dehydration. During data collection, the weights and heights of the students, environmental temperatures and air humidity levels were obtained.

**RESULTS:**

A total of 475 students aged six to 12 years were evaluated, of whom 188 were male. Voluntary dehydration occurred in 63.2% of the students and was more frequent in males than in females. The prevalence of voluntary dehydration was more frequent in males aged six to nine years than in females. However, no statistically significant difference was observed between males and females aged 10 to 12 years. No association was found between voluntary dehydration and nutritional status or socioeconomic status.

**CONCLUSION:**

The prevalence of voluntary dehydration was high in elementary school students and was more frequent in males. No association was found between voluntary dehydration and nutritional or socioeconomic status.

## INTRODUCTION

Voluntary dehydration occurs when the ideal volume of fluid required to meet an individual's needs is not ingested. Voluntary dehydration may be intensified in hot and dry environments and during physical activity 1-3. Importantly, voluntary dehydration should be distinguished from dehydration, which occurs secondary to abnormal fluid loss such as through vomiting and diarrhea.

In adults, voluntary dehydration can have harmful consequences on cognitive performance, including memory, attention, motor coordination and mood 1-5. Other symptoms that may be associated with voluntary dehydration in adults include fatigue, lack of appetite, drowsiness, thirst, discomfort in heat and reluctance to participate in complex tasks 1,4. In children, voluntary dehydration can alter visual attention, immediate memory, semantic flexibility, the ability to perform mathematical operations 1,2 and short-term memory 3. It can also cause thirst and discomfort in heat 4.

To diagnose voluntary dehydration, urinary biomarkers, particularly osmolarity of an isolated urine sample, can be used and are an excellent option due to the ease and practicality of sample collection, especially for studies in population groups 6-10.

Studies show a high prevalence of voluntary dehydration in groups of children and adolescents in various regions of the world 1-3,11-14. Voluntary dehydration is directly related to climate and sex; that is, it is more frequent in hot and dry climates 1-3,11 and among males 2,11-16. On the other hand, there is no relationship between voluntary dehydration and socioeconomic status 12,13, despite the existence of evidence showing greater fluid consumption by children of higher social classes 17.

In Brazil and Latin America, research on the prevalence of voluntary dehydration in healthy children is lacking. The relationship between voluntary dehydration and socioeconomic conditions, environmental temperatures and air humidity levels has not been previously studied in these countries.

Thus, the objective of the present study was to evaluate the prevalence of voluntary dehydration based on urine osmolarity in elementary school students in the metropolitan region of São Paulo. The study also evaluated whether there is a relationship between voluntary dehydration and socioeconomic status, environmental temperatures and air humidity levels.

## METHODS

### Design

This observational cross-sectional study was carried out in two public schools located in the city of Osasco, which is in the metropolitan region of São Paulo, Brazil. Our research team requested permission from the Secretary of Education of the Municipality of Osasco to conduct this study. Considering the number of students that should be included in the study, two elementary schools were chosen by the Secretary of Education. There are drinking fountains in both schools, which could be used by students during the intervals between classes. Furthermore, students are encouraged to bring a bottle of water to the classroom, and if necessary, students may leave the classroom to drink water.

A voluntary dehydration study was performed using urine osmolarity in an isolated urine sample; socioeconomic classifications, weight and height measurements and an evaluation of climatic conditions were also obtained on the day of the study. The study participants were enrolled after informed consent was given by the participants' caretakers and informed assent was provided by the participants. The data collection was initiated after the caretakers and participants signed the informed consent and assent forms, respectively. The study was approved by the Research Ethics Committee of the Universidade Federal de São Paulo (CEP 859.533).

Data were collected from November 2014 to November 2015. The main inclusion criterion of this convenience sample was age between six and 12 years old. Students with severe chronic diseases (genetic, neurological, cardiac, inflammatory, endocrine or renal) were not included in the study. Two students were not included, one with myelomeningocele and the other with cerebral palsy.

In total, 475 students were included in the study. Urine collection and weight/height measurements were performed in the morning (n=282) or afternoon (n=193), depending on the time of each student's class.

### Method

#### Voluntary dehydration protocol

Voluntary dehydration was defined according to urine osmolarity. A urine sample was collected from each participant in a sterile vial and stored at -20°C 1.

The urine sample analysis was carried out in the laboratory of the Hospital do Rim e Hipertensão (São Paulo, Brazil). The freezing method of the Advanced® Osmometer Model 3W2 (Advanced Instruments Inc., Massachusetts, USA) was used to determine urine osmolarity.

Voluntary dehydration was defined as osmolarity greater than 800 mOsm/kg H_2_O 1,3,12-14.

#### Meteorological data

Meteorological data (environmental temperatures and air humidity levels) were obtained from the Meteorological Database for Teaching and Research at the station closest to the municipality of Osasco, which was the Mirante de Santana station (Instituto Nacional de Meteorologia, Brazil). Relative air humidity values higher than 70% were considered adequate 18.

#### Economic classification

The Brazilian Economic Classification Criteria (Associação Brasileira de Empresas de Pesquisa- ABEP, São Paulo, Brazil) questionnaire was used for the economic classification.

#### Weight and height assessments

Weight and height measurements were evaluated according to the recommendations of the Food and Nutrition Surveillance System (Ministério da Saúde, Brasília, Brazil). Body mass index (BMI) was calculated by dividing the weight by the height^2^ (kg/m^2^).

Height-for-age (H/A) and BMI for age (BMI/A) scores were calculated using WHO AnthroPlus software version 1.0.4 (WHO, World Health Organization, Geneva, Switzerland). Nutritional status was classified according to the BMI/A, and the following cutoff points were adopted: z-score ≥-3 to <-2 for thin; z-score ≥-2 to ≤+1 for adequate weight; z-score >+1 to ≤+2 for overweight; z-score >+2 to ≤+3 for obese; and z-score >+3 for severely obese 19.

#### Statistical analysis

The sample size was calculated using the statistical program Epi-Info 6.04 (CDC, Centers for Disease Control and Prevention, Atlanta, Georgia, USA). A voluntary dehydration prevalence of 60% was expected based on the results of studies performed in other countries 1,2,11-14. The population of 40,000 school-age students in the municipality of Osasco was considered to have a sample error of 5% and a degree of confidence of 95%. Thus, it was estimated that a minimum sample size of 365 students would be required.

Parametric and nonparametric statistical tests were used to analyze the results according to the distributions of the variables. Statistica Ultimate Academic 12.7 (Statsoft Software, São Paulo, Brazil) and Sigma Plot 11.2 (Systat Software, San Jose, California, USA) software were used for the calculations.

For all tests, the level of significance was set at 5% or 0.05.

## RESULTS

A total of 475 students were evaluated, of whom 39.6% (N=188) were male and 60.4% (N = 287) were female. The mean ages of the boys and girls were 9.2±1.6 years and 9.1±1.4 years, respectively (*p*=0.389). A predominance of socioeconomic classes B2 (34.4%) and C1 (41.1%) was observed. Weight measures indicating overweight and obesity were found in 36.6% (N=174) of the studied population, with similar frequencies (*p*=0.253) in boys (33.5%, 63/188) and girls (38.7%, 111/287).

According to urine osmolarity measures, the prevalence of voluntary dehydration was 63.2% (300/475). The frequency of voluntary dehydration was very similar (*p*=0.614) in both the morning (62.1%, 175/282) and afternoon (64.8%, 125/193). Table 1 shows that the frequency of voluntary dehydration was higher in males than in females (71.8% and 57.5%, respectively, *p*=0.002). The prevalence of voluntary dehydration in 6- to 9-year-olds was more frequent in males (68.75%, 77/112) than in females (53.3%, 105/197, *p*=0.011). However, there was no statistically significant difference between males (76.3%; 58/76) and females (66.7%; 60/90, *p*=0.229) among 10- to 12-year-olds. The prevalence of voluntary dehydration was higher in 10- to 12-year-olds than in 6- to 9-year-olds, independent of sex. The prevalence of voluntary dehydration in males was similar between 6 and 9 years and between 10 and 12 years, whereas in girls, it was more frequent between 10 and 12 years (Table 1).

Table 2 shows that there was no relationship between voluntary dehydration and nutritional status in males or females.

Table 3 shows socioeconomic classes grouped into two categories: A2, B1 and B2 and C1, C2 and D, according to sex. There was no relationship between the occurrence of voluntary dehydration and socioeconomic class.

The mean environmental temperature was 21.2°C±3.8°C, and the mean air humidity level was 70.7%±17.0% in the twenty-one days during which urine collection was performed. A negative correlation was observed between the environmental temperature and the relative air humidity level (r=-0.77, *p*=0.000). The prevalence of voluntary dehydration on days when the environmental temperature was below and above the mean (21.2°C) were 61.4% (153/249) and 65.0% (147/226, *p*=0.474), respectively. The prevalence of voluntary dehydration on days with humidity levels lower and higher than 70% were 67.5% and 61.5% (*p*=0.221), respectively.

## DISCUSSION

This study is the first to evaluate the prevalence of voluntary dehydration in Brazil. Voluntary dehydration was observed in 63.2% of students included in the study between the ages of six and 13, and it was more frequent in males. No relationship was found between voluntary dehydration and nutritional status or socioeconomic status. The high prevalence of voluntary dehydration is compatible with a recent epidemiological survey that included Brazilian children and adolescents. It was observed that 47% of schoolchildren and 38% of adolescents presented daily fluid intake lower than the recommendation 20.

A study conducted with adult individuals showed that urinary osmolarity varies throughout the day 21. Urinary osmolality in the afternoon tends to be lower than in the morning 21. However, in our study, the prevalence of voluntary dehydration was similar in the morning and afternoon. One study carried out in Italy evaluating the effect of an intervention with a stimulus of water consumption during school activities showed that in the intervention group, the occurrence of voluntary dehydration decreased from 80% in the morning to 42% in the afternoon. In the control group, these values were 88% and 75%, respectively. This result showed that the prevalence of voluntary dehydration remained high during both periods of the day 3, similar to what was observed in our study.

The prevalence of voluntary dehydration in the present study was similar to that found in most studies conducted in other countries 1-3,11-14,22,23. The prevalence of voluntary dehydration in those studies, which used the same diagnostic criteria (urine osmolarity greater than 800 mOsm/kg H_2_O in an isolated sample of urine), varied between 33% and 84% 1-3,11-14,23. In Israel, the prevalence of voluntary dehydration ranged from 55.2% to 67.5% among children aged two to 12 years 1,2,11. Similar values were found in children aged nine to 11 years in Portugal 22, France 3 and the United States 14, at 58.5%, 62.2% and 66%, respectively. The highest prevalence was found in Italy, at 84% in children of the same age. On the other hand, the lowest prevalence (33%) was found in Greek children aged 9 to 13 years 23. Few studies have evaluated voluntary dehydration in healthy adults. In the United Kingdom, voluntary dehydration was observed in 19 (37%) out of 52 healthy adults before recreational physical activity 24. Another study, conducted with 573 healthy men and women from Spain, Greece and Germany, showed a prevalence of voluntary dehydration of 29% in the study population, occurring more frequently in men than women 25. In the US military, voluntary dehydration was observed in 33% of those studied and was more frequent among males 26. Thus, the available information indicates that voluntary dehydration is more frequent in the pediatric population.

In most studies 1,2,11-16,23, the prevalence of voluntary dehydration was higher in males. This difference may be related to behaviors around fluid consumption. In Germany, a study evaluated 495 healthy children and observed that girls may have a preference for foods with higher water densities and have lower insensible water losses compared with boys 15. Another factor that may explain the higher prevalence of voluntary dehydration in boys is the higher physiological needs of the male sex 13.

In our study, as in those conducted in Massachusetts and France, no relationship was found between socioeconomic status and voluntary dehydration 12,13. On the other hand, in Guatemala, children in the upper social classes consumed larger volumes of water than children in the lower classes 17; however, that study did not evaluate the occurrence of voluntary dehydration.

A high prevalence of voluntary dehydration may be related to warmer and drier climates 1,2. In our study, voluntary dehydration was more frequent on warmer days and on days with lower air humidity levels, but the effect was not statistically significant. However, in our study, the air humidity levels on collection days were similar in all seasons.

Our study has some limitations. The study was performed in only one city and thus does not represent the entire Brazilian population. The results of the present study justify the development of future projects in not only public but also private schools. This strategy will allow the inclusion of samples with wider variations in family incomes. Moreover, our research group is developing a project to link voluntary dehydration and intestinal constipation in the pediatric age group 27,28.

In conclusion, the prevalence of voluntary dehydration was high in primary school students and was more frequent among male students between 6 and 9 years of age. Children between 10 and 12 years of age are at increased risk for voluntary dehydration independent of sex. There was no association between voluntary dehydration and socioeconomic status or nutritional status.

## AUTHOR CONTRIBUTIONS

Dias FC conceived the research, collected the data, interpreted the collected data, wrote the manuscript and approved the final version of the manuscript. Boilesen SN, conceived the research, collected the data and approved the final version of the manuscript. Melli LCFL and Tahan S conceived the research, interpreted the collected data, reviewed the manuscript and approved the final version of the manuscript. Morais MB conceived the research, interpreted the collected data, wrote the manuscript and approved the final version of the manuscript.

## Figures and Tables

**Table 1:** Voluntary dehydration in students from two public schools in the metropolitan region of São Paulo, according to sex.

	Voluntary dehydration			
	Yes (>800 mOsm/kg H_2_O)	No (≤800 mOsm/kg H_2_O)	*p*^a^	Odds ratio IC 95%
6 to 9 years				
Males	77 (68.75%)	35 (31.25%)		
Female	105 (53.3%)	92 (46.7%)	0.011	1.9 (1.2;3.2)
Total	182 (58.9%)	127 (41.1%)		
10 to 12 years				
Males	58 (76.3%)	18 (23.7%)		
Female	60 (66.7%)	30 (33.3%)	0.229	1.6 (0.8;3.2)
Total	118 (71.1%)	48 (28.9%)		
All Students				
Males	135 (71.8%)	53 (28.2%)		
Female	165 (57.5%)	122 (42.5%)	0.002	1.9 (1.3;2.8)
Total	300 (63.2%)	175 (36.8%)		

a Pearson’s Chi-square test.

**Table 2:** Voluntary dehydration in students from two public schools in the metropolitan region of São Paulo, according to nutritional status and sex.

		Voluntary dehydration		
	Nutritional status	Yes (>800 mOsm/kg H_2_O)	No (≤800 mOsm/kg H_2_O)	*p*^a^
Male	Thin	3 (100.0%)	0 (0.0%)	
	Adequate weight	88 (72.1%)	34 (27.9%)	
	Overweight	19 (61.3%)	12 (38.7%)	0.475
	Obese	21 (77.8%)	6 (22.2%)	
	Severely obese	4 (80.0%)	1 (20.0%)	
Female	Thin	1 (50.0%)	1 (50.0%)	
	Adequate weight	96 (55.2%)	78 (44.8%)	
	Overweight	39 (61.9%)	24 (38.1%)	0.156
	Obese	27 (65.9%)	14 (34.1%)	
	Severely obese	2 (22.2%)	7 (77.8%)	

a Pearson’s Chi-square test.

**Table 3:** Voluntary dehydration in students from two public schools in the metropolitan region of São Paulo, according to socioeconomic classification and sex.

		Voluntary dehydration		
	Socioeconomic classification	Yes (>800 mOsm/kg H_2_O)	No (≤800 mOsm/kg H_2_O)	*p*^a^
Male	A2, B1, B2	56 (76.7%)	17 (23.3%)	0.216
	C1, C2, D	73 (68.2%)	34 (31.8%)	
Female	A2, B1, B2	62 (54.4%)	52 (45.6%)	0.378
	C1, C2, D	95 (59.7%)	64 (40.3%)	

aPearson’s Chi-square test.
